# Data set for influence of blends of diesel and renewable fuels on compression ignition engine emissions

**DOI:** 10.1016/j.dib.2019.104836

**Published:** 2019-11-18

**Authors:** A.S. van Niekerk, B. Drew, N. Larsen, P.J. Kay

**Affiliations:** University of the West of England, Coldharbour Lane, Bristol, BS16 1QY, UK

**Keywords:** Biodiesel, Ethanol, Engine emissions, Ternary blend, Design of experiment, WLTP

## Abstract

The present data article is based on the research work which investigates the influence of blends of diesel and renewable fuels on compression ignition engine emissions. In this experimental work, a 2.4 L, turbocharged, direct injection compression ignition engine and water brake dynamometer were used. Different ternary blends were created by mixing diesel, biodiesel and ethanol together in accordance with a mixture design of experiments. The homogeneity of each ternary blend was qualitatively checked by observing the samples for 24 hours for visible separation.

The engine was run over the WLTP drive cycle for each individual ternary blend and the exhaust emissions were recorded. NOVA 7466K and TESTO 350 gas analysers were used to record the exhaust emissions. A factory standard MAF sensor was used to record the inlet air mass flow and an aftermarket ECU was used to determine the fuel flow. The ternary blends were blended using standard laboratory measuring equipment.

**Table:** Specifications Table

Subject	Automotive Engineering
Specific subject area	Ternary blends with renewable fuels reducing harmful emissions
Type of data	Images, tables and computer code
How data were acquired	Experimental emissions analysis in engine testing facility
Data format	Raw and tabulated
Parameters for data collection	Each test was conducted over the WLTP drive cycle, with the fuel composition as the only changing parameter. The composition of the fuel consisted of a ternary blend between diesel, biodiesel and ethanol. The percentages of each blend component were varied for each test.
Description of data collection	Engine emissions data were collected with exhaust gas analysers, intake air flow was recorded with a mass airflow sensor, fuel flow values were recorded from an aftermarket ECU.
Data source location	University of the West of England, Bristol, United Kingdom
Data accessibility	With the article
Related research article	A.S. van Niekerk, B. Drew, N. Larsen, P.J. Kay Influence of blends of diesel and renewable fuels on compression ignition engine emissions over transient engine conditions Applied Energy, https://doi.org/10.1016/j.apenergy.2019.113890

**Table:** 

**Value of the Data** • This data set contains emissions information from ternary blends of renewable fuel. Detail emission information is critical for the development of future automotive technologies to reduce on-road emissions. • The data will be useful for a range of beneficiaries. Researchers in the same discipline (automotive/mechanical engineering), researchers in different disciplines such as air quality and transportation; the information may also be useful to non-technical beneficiaries such as all party parliamentary groups. • The data can be used in a number of ways, such as to validate engine combustion models, vehicle emission models and simple transport models. • The data can be used to support policymakers in determining a feasible alternative to achieve a specific renewable content in transport energy while reducing harmful emissions.

## 1 Data

The data presented in this article were based on the experimental study on the influence of diesel and renewable fuels on emissions of compression ignition engines. Table 1 represents the data regarding fuel properties of diesel, biodiesel and ethanol as provided by the fuel supplier. Table 1 also include the fuel properties of all the binary and ternary blends used in the research. The experimental set up investigated blends between diesel, biodiesel and ethanol ranging from pure diesel (D100) to binary blends with biodiesel or ethanol (B20, E20) and ternary blends (B14E3, B7E7 etc.). The ‘B’ denotes the percentage of biodiesel in the blend by volume. Similarly, the ‘E’ denotes the percentage of ethanol in the fuel blend by volume.

**Table 1:** Physiochemical properties of diesel, biodiesel, ethanol and their blends.

	Cetane number	LHV (MJ/kg)	Density at 15 °C (kg/m^3^)	Viscosity at 40 °C (mm^3^/s)	CFPP (°C)	Flash point (°C)
Diesel	51.7	42.8	831.1	2.686	−26	65
Biodiesel	52.8	39.0	883.2	4.372	−6	179
Ethanol	7.0	26.8	790.0	1.200	−38	40
B20	51.9	41.8	841.5	3.023	−22	87
E20	42.8	39.6	822.9	2.388	−28	60
B14E3	50.5	41.6	837.2	2.877	−24	80
B3E14	45.5	40.4	826.9	2.529	−27	65
B3E3	50.4	42.2	831.4	2.692	−26	67
B7E7	48.7	41.3	831.9	2.700	−25	71

The engine used for the data collection is a 2.4 L Euro IV turbocharged compression ignition engine with an aftermarket ECU. The engine parameters are listed in Table 2. The measurement of the exhaust gasses was conducted using two gas analysers; one (NOVA 7466K) for measuring CO_2_ emissions and NO_x_ emissions and the other (TESTO 350) for measuring CO emissions. This was done to ensure the highest accuracy in the measurement, as the two gas analysers have different accuracy levels for different exhaust gasses. Both were located upstream of any exhaust after treatment systems. A summary of the analyser accuracy and measurement method is presented in Table 3.

**Table 2:** Engine parameters used for experimentation.

Engine parameter	Characteristics
Bore (mm)	89.9
Stroke (mm)	94.6
Volume (cc)	2402
Compression ratio (CR)	17.5
Number of cylinders	4
Method of cooling	Water cooled

**Table 3:** Method and accuracy of the instruments used to measure the engine emissions.

Exhaust gas	Range	Accuracy	Method
CO (ppm)	0–10000	<10	electrochemical
CO_2_ (ppm)	0–20	<20	infra-red
NO (ppm)	0–2000	<2000	electrochemical
NO_2_ (ppm)	0–800	<8	electrochemical

The factory fitted mass airflow sensor (MAF) was calibrated with a Superflow SF-120 flow bench and was used to measure the intake mass air flow.

The fuel consumption was determined by weighing the fuel tank before and after the test run and dividing the difference by the distance travelled over the WLTP. A Kern EMB 5.2K1 scale was used to weigh the fuel tank with an accuracy of ±1g.

The experimental response of 14 runs in the design matrix along with their corresponding points of the fitted mixture design are shown in Table 4. All 14 runs were cold start runs, with the engine oil and cooling water temperature at approximately 20 °C (σ = 2) at the start of each test.

**Table 4:** The experimental values of the engine response for the mixture DoE.

Run	Parameter settings			Experimental response (g/km)			
	x_D_	x_B_	x_E_	CO	CO_2_	NO_x_	fc
1	0.80	0.00	0.20	2.0598	244.52	1.0812	134.00
2	0.83	0.14	0.03	1.2046	258.74	1.0841	122.75
3	0.83	0.03	0.14	1.4141	232.37	1.0584	128.75
4	0.86	0.07	0.07	1.1409	251.43	1.0566	119.27
5	1.00	0.00	0.00	1.0182	252.03	1.1148	120.64
6	0.83	0.14	0.03	1.0624	257.02	1.0841	121.11
7	0.80	0.20	0.00	0.8844	238.07	1.0669	123.23
8	0.83	0.03	0.14	1.2837	232.37	1.0370	129.98
9	0.94	0.03	0.03	0.9863	244.98	1.0741	121.79
10	0.80	0.20	0.00	0.8868	247.06	1.0714	126.50
11	0.94	0.03	0.03	0.9724	247.48	1.0948	126.57
12	1.00	0.00	0.00	0.9874	251.08	1.1518	116.41
13	0.86	0.07	0.07	1.0638	241.27	1.0635	121.18
14	0.80	0.00	0.20	1.9894	245.00	1.0764	135.50

## 2 Experimental design, materials, and methods

In order to ensure that the old fuel blend from the previous test does not influence the next test, the fuel system was flushed with the next test's blend of fuel before formal testing began.

It was necessary to determine the amount of flushes required that will successfully remove all remaining fuel blend from the previous test. This was done using red fuel dye and conventional diesel fuel. A sample of the clean diesel fuel was photographed using a Canon EOS 700D under homogeneous light conditions. After the dye was added to the fuel tank, the following procedure was followed using the engine's fuel delivery system:

Step 1: Run engine with fuel which contains the red dye.

Step 2: Use fuel primer pump to pump out all fuel from the fuel system.

Step 3: Replace current fuel filter with an empty fuel filter.

Step 4: Replace fuel in the fuel tank with clean fuel and run the fuel primer pump for 5 minutes.

Step 5: Idle engine for 5 minutes.

Step 6: Run engine at 2500 rpm for one minute.

Step 7: Use fuel primer pump to pump out all the fuel from the fuel system.

A sample of the fuel in the fuel tank was taken after the steps were followed and a photo was taken of the fuel sample. Steps 3–7 were then repeated and another photo was taken of the fuel sample. After sufficient iterations were performed, the image files were imported to Matlab:

*Img_base = imread('base.jpg');*
*Img_i = imread('i.jpg');*

where *base* refers to the image of the fuel sample with no dye added to it and *i* refers to the fuel sample taken after each iteration of the engine being flushed with clean fuel. A rectangular area was then determined that has a uniform distribution of colour for all the images. The coordinates of the rectangle were inputted as a column matrix into the software. New image files were created by cutting out the rectangle from the original photos of the fuel samples:

*Img_base_crop = imcrop(Img_base,rectangle);*
*Img_crop_i = imcrop(Img_i,rectangle);*

After the new images were created, the red spectrum of the base fuel's cropped image (clean fuel sample) was subtracted from the red spectrum of each iteration's cropped image. This was done to eliminate any red colour that is present in the clean fuel sample. As a result the red spectrum present in the cropped image is that of the fuel dye only.

*Img_final_i = Img_crop_i (:,:,1) - Img_base_crop (:,:,1);*

The red spectrum present in the fuel samples after each flush iteration was shown visually with a histogram. The histogram graphs were used qualitatively to determine after which flush the majority of the red dye has been removed. This can be seen when the histogram shows that the majority of the red spectrum equals zero (no red colours present in that pixel).

**Fig. 1:**
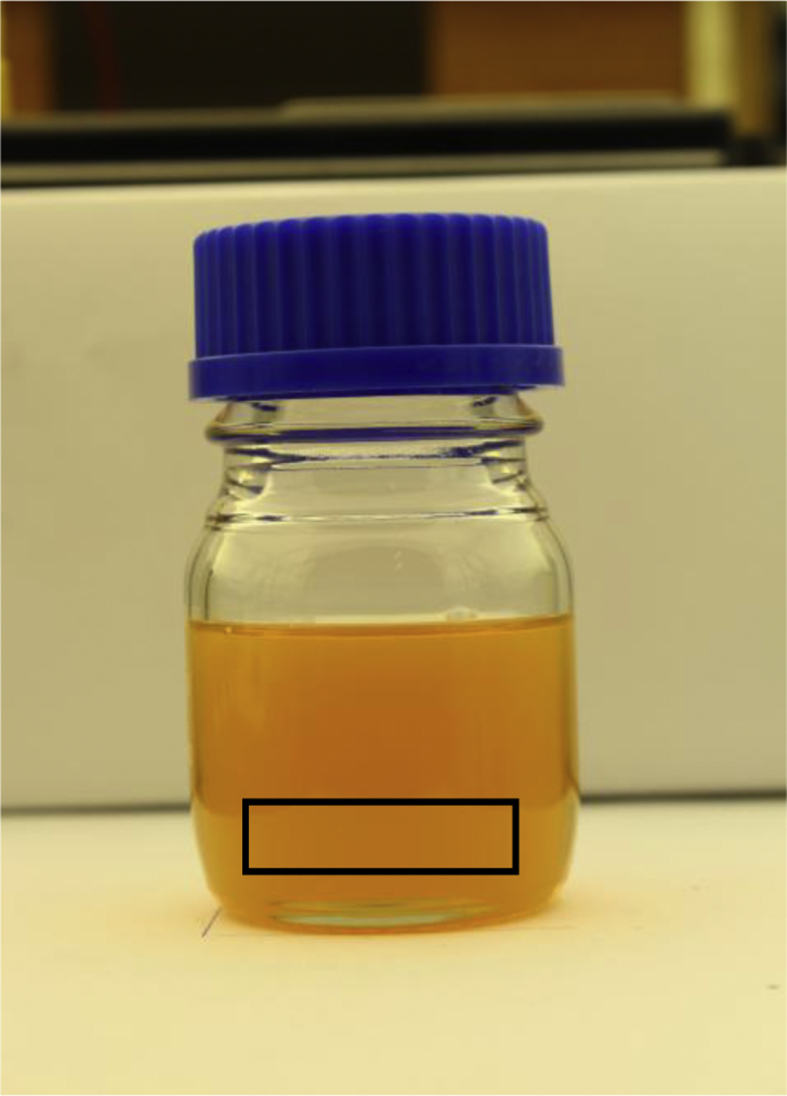
Visual representation of area used to calculate the red spectrum distribution for each fuel sample [1].

It is important to ensure that when taking pictures of the fuel samples, the camera is mounted on a tripod and all samples are placed in the same position to ensure that the photographs of the samples were the same size for accurate comparisons to be made. This enables accurate comparison between cropped areas of the photos taken of the fuel samples as seen in Fig. 1.

The blend ratios as seen in Table 4 were determined using Minitab, a statistical software program. For a ternary blend experimental set up the following equations need to be true:

(1) $$0<x_{i}<1,i=D,B,E$$

and

(2) $$x_{D}+x_{B}+x_{E}=1$$

where $x_{D},$ $x_{B},$ and $x_{E}$ refer to diesel, biodiesel and ethanol proportions in the blend. For this research upper limits for biodiesel and ethanol were set at $x_{B},x_{E}\leq 0.2$ to ensure that no engine modification is needed to run the blends [2,3]. Centroid and axial points have been added in the statistical software to increase the capability of the software to fit quadratic equations for ternary blends. The mixture design was replicated once and the runs were randomised to ensure the experimental errors were independently distributed.
